# Origin of nonlinear force distributions in a composite system

**DOI:** 10.1038/s41598-021-04693-8

**Published:** 2022-01-12

**Authors:** Yuto Tamura, Marie Tani, Rei Kurita

**Affiliations:** Department of Physics, Tokyo Metropolitan University, 1-1 Minamioosawa, Hachiouji-shi, Tokyo 192-0397 Japan

**Keywords:** Soft materials, Structural materials, Composites

## Abstract

Composite materials have been actively developed in recent years because they are highly functional such as lightweight, high yield strength, and superior load response. In spite of importance of the composite materials, mechanisms of the mechanical responses of composites have been unrevealed. Here, in order to understand the mechanical responses of composites, we investigated the origin and nature of the force distribution in heterogeneous materials using a soft particle model. We arranged particles with different softness in a lamellar structure and then we applied homogeneous pressure to the top surface of the system. It is found that the density in each region differently changes and then the density difference induces a nonlinear force distribution. In addition, it is found that the attractive interaction suppresses the density difference and then the force distribution is close to the theoretical prediction. Those findings may lead material designs for functional composite materials.

## Introduction

Mechanical properties of single component materials are expressed by typical parameters such as Young’s modulus and Poisson’s ratio and those parameters are important for material designs, construction industries and so on. On the contrary of the single component materials, the mechanical properties of composites, which are substances of combining multiple materials, cannot be described by linear combination of those parameters of each component^1,2^. Macroscopic material properties depend on the internal structure or arrangement of the components. Thus, the composite materials with designed arrangement, which is sometimes called a metamaterial, have superior functions such as high strength despite lightness, toughness and highly fracture resistance exceeding the simple combinations^3–9^. Those composites can be well observed in nature and biology. For an example, pearl shells have lamella structure of hard and soft layers and the soft layers absorb the energy of the cracks and then the material has resistant to fracture^10^. Recently, the composite materials mimicked the biomaterial, which is called biomimetics, have been developed^11–13^. Then the composite materials have been put to practical uses in airplanes and cars by realizing weight reduction and improving heat resistance^14,15^. Therefore, development of novel composite materials is actively promoted and the elucidation of their physical properties is expected.

In order to design the composites, mechanical responses for the external force in the composites are crucial. The mechanical properties are strongly connected with the force distribution inside the material. For example, the external force locally propagates like a chain in granular materials and it is related with the unique feature of the granular materials^16^. The jamming systems, which are densely packed particles, such as colloidal dispersion systems^17^, foams^18–20^ and emulsions^21^ also show the chain-like force propagation and then they show peculiar phenomena such as relaxation^22^, shear thickening, and shear thinning^23–25^. Particle simulations are being actively performed on such systems, and it has been found that these phenomena are caused by slight changes such as the change of the number of contact particles and the rearrangement of the internal structure. Although the mechanical properties in the single component systems have been investigated, the mechanism of the distribution of the external force in the composites has been unclear.

Here, understanding the force distribution mechanism can become a base of composite material design such as mixing method and mixing ratio and it will greatly lead to industrial developments in future. In this study, we focus on the non-uniformity of elastic modulus inside the material, and perform a numerical simulation using repulsive soft particles with different softness. The purpose is to clarify the mechanism of the nonlinear distribution of the force.

## Model and methods

**Figure 1:**
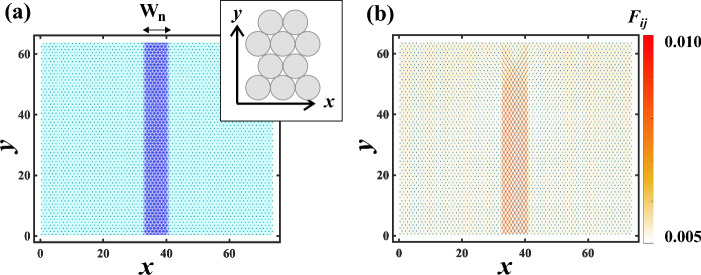
(**a**) $k_{ij}$ distribution. Dark color corresponds to $k_{ij}$ = 3, while bright color corresponds to $k_{ij}$ = 1. $W_n$ is a width of G$_h$ region. The inset schematic shows the arrangement of the triangular lattice. (**b**) Magnitude of the force is represented by the color, as indicated by the color bar. The force is larger in G$_h$ region.

In this study, we investigated a two-dimensional binary repulsive soft particle model where the softness of each particle is different. We consider this system as a simple model of a composite elastic body with binary components. We arranged 4096 soft disks in a 64 $\times$ 64 triangular grids. We set orientation of the triangular lattice so that the base of the triangle is parallel to the *x* axis in order that plastic rearrangement events hardly occur by the external force in *y* axis (see an inset of Fig. 1a). The periodic boundary condition is applied in the *x* direction and the wall is located at *y* = 0. The particles are arranged at $y>$ 0.

Pairs of the disks *i* and *j* interact via the pairwise harmonic repulsive potential:

1 $$\begin{aligned} U(r_{ij}) = \frac{k_{ij}}{2} (r_{ij}-D)^2 f(D-r_{ij}) \end{aligned}$$

where $k_ {ij} = (G_i + G_j) / 2$, where $G_i$ is a parameter corresponding to the softness of the disk *i*. $r_{ij}$ is a center-to-center distance between disks *i* and *j*, and *D* is a diameter of disks. $f(r) = k_{att}$ for $r < 0$ and $f(r) = 1$ for $r > 0$, where $k_{att}$ is a positive constant and it corresponds to the strength of the attractive interaction. $k_{att}$ = 0 when the interaction is only repulsive. In this model, the overlap between pairs of particles is allowed and the friction between the disks is neglected for simplicity. We set $n_x$ and $n_y$ as a number of the layer from the origin in *x* direction and *y* direction, respectively. Here we normalized the length by the initial distance of the layers in *y* direction. Thus, before applying the external force, *xy* coordinate of the disk for $n_x$ and $n_y$ is equal to $(x, y) = (n_x/\sqrt{3}, n_y)$.

We investigated a lamella structure in which the region of soft disks (G$$_s$$ region) and the region of hard disks (G$$_h$$ region) are arranged parallel to *x* axis (Fig. 1a). We fix $$G_i$$ = 1 for the softer disk and we define $$G_i = G_h$$ for the harder disk. The width of G$$_h$$ region $$W_n$$ is set to $$W_n$$ = 4, 6, or 8. The change of $$W_n$$ corresponds to that of the area ratio between G$$_s$$ region and G$$_h$$ region. We added $$F_{ex} = -0.01$$ in the *y* direction to the disks located at the top, that is, the external force direction is perpendicular to the lamella structure. The strength of $$F_{ex} $$ corresponds to the force when the size of the softer disk with $$G_i$$ = 1 shrinks by 1 %. Each particle moves following the normalized overdamped equation.

2 $$\begin{aligned} 0 = \frac{d r_i}{dt} - \frac{\partial U(r_{ij})}{\partial r}, \end{aligned}$$

where $r_i$ is a position of disk *i*. The repulsive normal force between the disk *i* and the bottom wall is $F_N = (D/2 - y_i)(G_w + G_i)/2$ when $y_i < D/2$. We set $G_w = 1000$. When the maximum velocity in all particles becomes less than 1.0$\times$10$^{-6}$, we regard that the system reaches a steady state.

## Force distribution in a repulsive system

Firstly, we investigated the force distribution in the repulsive system ($k_{att} = 0$) with $G_h = 3$. Figure 1b shows the force distribution represented by the color at $W_n = 8$ and then it is found that the force is larger in the G$_h$ region than in the G$_s$ region. It was also seen that the force distribution in the upper region is different from that in the lower region due to the influence of the boundary condition of the top surface. Here, we focus on the properties of the bulk and then we analyzed the lower region.

**Figure 2:**
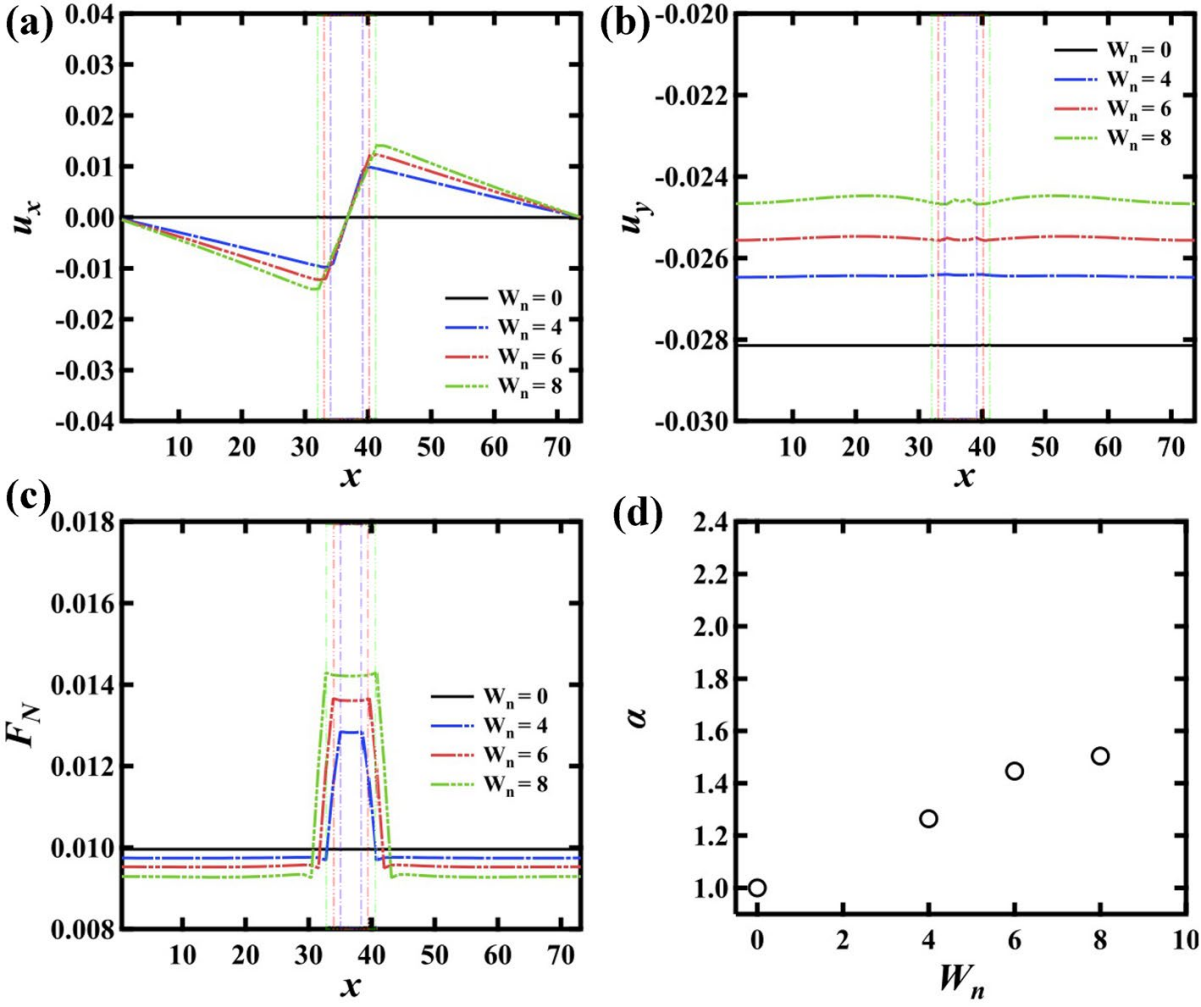
(**a**) Displacement in the *x* axis direction $$u_{x}$$ and (**b**) displacement in the *y* axis direction $$u_y$$ at $$n_{y} = 12$$. The vertical dashed lines represent the interfaces between G$$_h$$ region and G$$_s$$ region. $$u_{x} $$ has a positive correlation with *x* in the G$$_h$$ region. Meanwhile, $$u_{y}$$ is almost constant. (**c**) The normal force $$F_N$$ on the bottom surface. (**d**) The ratio of the normal stress at G$$_h$$ region to that at G$$_s$$ region $$\alpha $$. $$\alpha $$ increases with increasing $$W_n$$.

We also computed the displacement of the particles in *x* direction $$u_{x}$$ and in *y* direction $$u_{y}$$ at $$n_y$$ = 12 as a function of *x* with $$G_h = 3$$. Figure 2a,b show $$u_x\,u_y$$ for $$W_n$$ = 4 (blue line), 6 (red line), and 8 (green line), respectively. It is found that $$u_{x}$$ shows a characteristic change. $$u_{x} $$ has a positive correlation with *x* in the G$$_h$$ region, while $$u_{x}$$ has a negative correlation in the G$$_s$$ region. It means that the particles at the interface between the G$$_s$$ region and the G$$_h$$ region are pushed toward the G$$_s$$ region because $$k_{ij}$$ is asymmetric at the interface. Therefore, the density in the G$$_s$$ region becomes larger than in the G$$_h$$ region. It is also found that the slopes of $$u_{x}$$ subtly decreases in the G$$_h$$ region when $$W_{n}$$ increases (0.0039 for $$W_n$$ = 4 and 0.0031 for $$W_n$$ = 8). Meanwhile, $$u_{y}$$ is less dependent on the value of *G* although the constant external force is applied, not the external control of the displacement in *y* direction. We note here that the subtle difference of $$u_y$$ in *x* direction depends on $$n_y$$, thus this comes from an effect of the top surface boundary. However, $$u_y$$ can be regarded as constant macroscopically. In addition, $$u_y$$ becomes larger with increasing $$W_n$$.

Then, we examined the normal force $F_N$ on the bottom surface. It is found that the normal force in G$_h$ region becomes stronger than that in G$_s$ region (Fig. 2c). We define $\alpha$ as the ratio of the normal stress at $n_x$ = 32 to that at $n_x$ = 0. Figure 2d shows $\alpha$ as a function of $W_n$. It is found that $\alpha$ slightly increases with increasing $W_n$. We will discuss the mechanism for the force distribution later.

**Figure 3:**
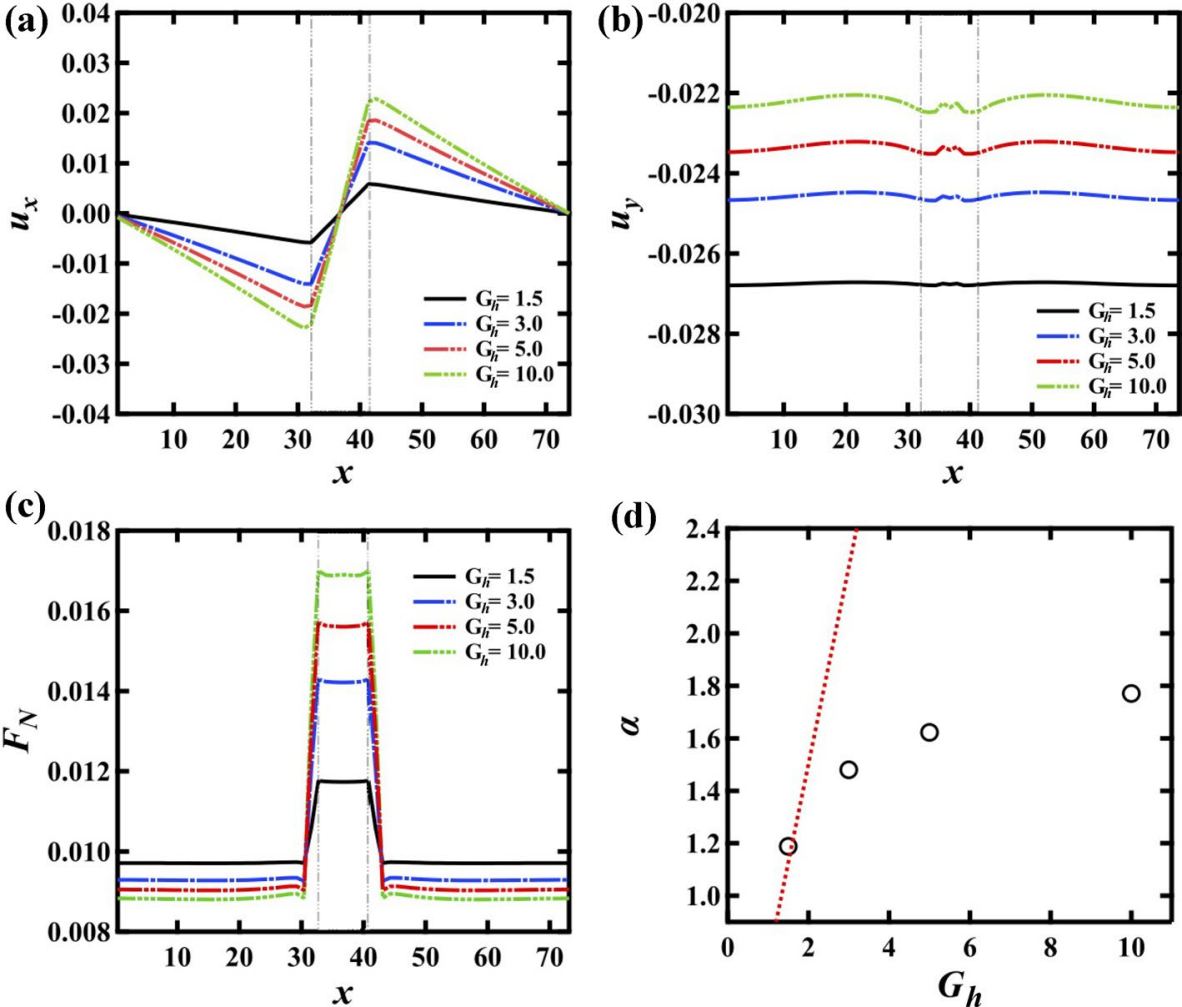
(**a**) Displacement in the *x* axis direction $u_{x}$ and (**b**) displacement in the *y* axis direction $u_y$ at $n_{y} = 12$. (**c**) The normal force $F_N$ on the bottom surface. (**d**) The ratio of the normal stress at G$_h$ region to that at G$_s$ region $\alpha$. The dotted line in (**d**) corresponds to the theoretical prediction using the continuum approximation. $\alpha$ is much less than the theoretical prediction. This deviation comes from the density change of G$_h$ region.

Next, we investigated the force distribution by changing $G_h$ between $G_h$ = 1.5 and 10. Figure 3a,b show $u_x\,u_y$ at $W_n = 8$ and $n_y$ = 12 for $G_h$ = 1.5 (black line), 3 (blue line), and 5 (red line), and 10 (green line), respectively. The positive relation of $u_x$ in the G$_h$ region becomes stronger when $G_h$ increases. It is also found that $u_y$ increases with increasing $G_h$. We also note that the difference of $u_y$ in *x* direction becomes larger with increasing $G_h$. We confirmed that the effect of the top surface is also stronger when $G_h$ is larger. Therefore, we conclude that the subtle difference of $u_y$ in *x* direction is caused by the effect of the top surface boundary. However, the difference is still small and thus $u_y$ can be regarded as constant macroscopically. Figure 3c,d show $F_N$ on the bottom surface and $\alpha$, respectively. The dotted line in (d) corresponds to the theoretical prediction using the continuum approximation, which we discuss later. It is found that $\alpha$ increases only from 1.2 to 1.8 and this is much less than the theoretical prediction.

## Force distribution in an attractive system

**Figure 4:**
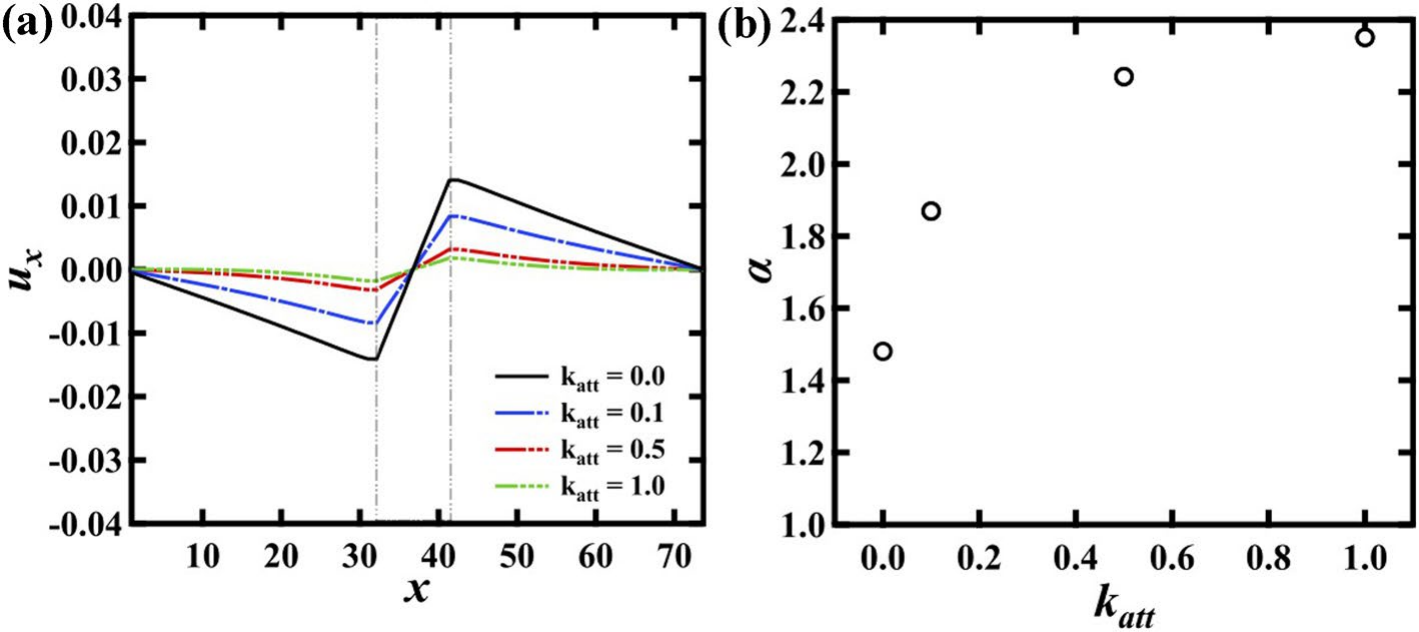
(**a**) Displacement in the *x* axis direction $u_{x}$ at $n_{y} = 12$ with the attractive interaction systems. $G_h$ = 3 and $W_n$ = 8. (**b**) $\alpha$ as a function of $k_{att}$. When the attractive interaction becomes stronger, $u_x$ becomes flat and then $\alpha$ becomes close to the theoretical prediction.

Furthermore, we investigate the force distribution with the attractive interaction. Figure 4a shows $u_x (x)$ with changing the spring constant $k_{att}$ at $W_n$ = 8 and $G_h$ = 3. When $k_{att}$ becomes larger, the gradient of $u_x$ in G$_h$ region becomes smaller. It is natural that the attractive interaction suppresses the separation of the particles. Figure 4b shows $\alpha$ with respect to $k_{att}$. It is found that $\alpha$ becomes larger for larger $k_{att}$. Here we note that $\alpha$ becomes close to the theoretical prediction (2.4 for $G_h$ = 3) at $k_{att} = 1$.

From $W_n$ and $k_{att}$ dependence of $\alpha$ (Figs. 2d, 4b), it is found that $\alpha$ becomes close to the theoretical prediction when the gradient of $u_x$ in G$_h$ region is small. In addition, the deviation of $\alpha$ from the theoretical prediction is large for larger $G_h$. Meanwhile, the slope of $u_x$ in the G$_h$ region is larger for larger $G_h$, smaller $W_n$, and smaller $k_{att}$. It suggests that the deviation of $\alpha$ from the theoretical prediction should be correlated with the slope of $u_x$ in the G$_h$ region.

## Origin of the nonlinear force distribution

**Figure 5:**
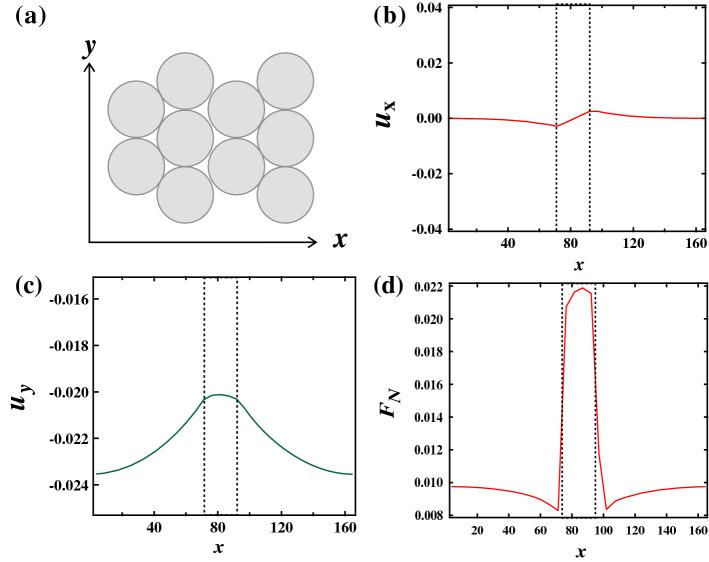
(**a**) Schematic of the arrangement of the triangular lattice. (**b**) Displacement in the *x* axis direction $u_{x}$ and (**c**) displacement in the *y* axis direction $u_y$ at $n_{y} = 12$. (**d**) The normal force $F_N$ on the bottom surface.

Here we discuss the reason why $u_y$ is almost constant even though the softness is inhomogeneous. At the interface between G$_s$ and G$_h$ regions, the particles in the G$_s$ region and the particles in the G$_h$ region are staggered. The particles at the interface are geometrically pinned with respect to the movement in *y* direction and then $u_{y}$ should be continuous at the interface. Since the structural deformation is small, $u_{y}$ is almost independent of $G_i$. In order to check the pinned effect of the staggered arrangement, we examine the system where the orientation of the triangular lattice is rotated by 30 degrees, that is, the base of the triangle is parallel to the *y* axis. We investigate displacements and force distribution at $W_n$ = 8 and $G_h$ = 3. It is found that $u_x$ is almost constant and $u_y$ is large in the G$_h$ region (see Fig. 5). The geometrical binding becomes much weaker in *y* direction and then the force propagates straightly in *y* direction. Then the particles mainly move in *y* direction rather than in *x* direction. Thus, we confirm that the staggered interface induced that $u_y$ is almost constant with respect to *x*.

Then we compare the continuum approximation in our system. We assume an isotropic elastic body where the height is *H*. The width of G$_s$ region is $W^{(s)}$ and the width of G$_h$ region is $W^{(h)}$ ($W^{(h)} \ll W^{(s)}$). We also set the interface between G$_s$ region and G$_h$ region is bound. This assumption represents the geometric bound interface in our simulation. It is known that $P^{(a)}_{ij} = \lambda ^{(a)} \sum _l E^{(a)}_{ll} \delta _{ij} + 2\mu ^{(a)} E_{ij}$, where *i*, *j*, and *l* are *x* or *y* and the superscript *a* is *s* for G$_s$ region or *h* for G$_h$ region. $P^{(a)}_{ij}$ and $E^{(a)}_{ij}$ are the stress tensor and the strain tensor in $G_a$ region, respectively. The sign of $P^{(a)}_{ij}$ and $E^{(a)}_{ij}$ is defined as positive when the direction is from the inside to the outside of each region. $\lambda$ and $\mu$ are the Lamé’s constant. Young’s modulus *Y* and Poisson’s ratio $\sigma$ can be described as $Y = \mu (3\lambda +2\mu )/(\lambda +\mu )$ and $\sigma = \lambda /2(\lambda +\mu )$, respectively. To simplify, we apply the constant deformation $HE_{yy}$ in *y* direction, not the constant pressure. Then the deformation in *x* direction is described as $W^{(h)} E^{(h)}_{xx} = -W^{(s)} E^{(s)}_{xx}$. Since the force in *x* direction is balanced at the interface, we obtained $P^{(s)}_{xx} = P^{(h)}_{xx}$. Here we assume that $Y^{(h)} = k Y^{(s)}$, and the Poisson’s ratio is same. It leads $\lambda ^{(h)} = k \lambda ^{(s)}$ and $\mu ^{(a)} = c \lambda ^{(a)}$, where $c = (1-2\sigma )/2\sigma$. Then we obtain

3 $$\begin{aligned} E^{(h)}_{xx} = -\frac{k-1}{(1+2c)(k+\gamma )}E_{yy} \end{aligned}$$

where $$\gamma = W^{(h)}/W^{(s)}$$. Then

4 $$\begin{aligned} P^{(s)}_{yy}= & {} \lambda ^{(s)} \left[ \frac{\gamma (k-1)}{(k+\gamma )(1+2c)}+1+2c \right] E_{yy} \end{aligned}$$

and

5 $$\begin{aligned} P^{(h)}_{yy}= & {} k \lambda ^{(s)} \left[ \frac{-k+1}{(k+\gamma )(1+2c)}+1+2c \right] E_{yy}. \end{aligned}$$

Finally, we obtain

6 $$\begin{aligned} \alpha = \frac{P^{(h)}_{yy}}{P^{(s)}_{yy}} = k\frac{(1+2c)^2(k+\gamma )-k+1}{(1+2c)^2(k+\gamma )+\gamma (k-1)}. \end{aligned}$$

Meanwhile, the density can be described as below;

7 $$\begin{aligned} \rho ^{(h)}= & {} \frac{\rho _0}{(1+E_{yy})(1+E^{(h)}_{xx})} \end{aligned}$$

8 $$\begin{aligned} \rho ^{(s)}= & {} \frac{\rho _0}{(1+E_{yy})(1-\gamma E^{(h)}_{xx})} \end{aligned}$$

where $\rho _0$ is a density before the deformation. Here we assume $\gamma \approx 0$ since $W^{(h)} \ll W^{(s)}$. We also set *c* is constant because the Poisson’s ratio weakly depends on the materials ($0< \sigma < 0.5$). Then it is obtained that $E^{(h)}_{xx} = -(k-1)E_{yy}/(1+2c)k$, $\alpha = [4c(1+c)k+1]/{(1+2c)^2}$, and $\Delta \rho = \rho ^{(s)}-\rho ^{(h)} = \rho _0 E^{(h)}_{xx} /(1+E_{yy})(1+E^{(h)}_{xx})$. If *k* is independent of the density, *k* should be same as the ratio of *G*. Here $\sigma$ is approximately 1/3 for metals and then we obtained *c* = 0.5. The dotted line in Fig. 3d is a prediction at *c* = 0.5. It is found that $\alpha$ obtained by the simulation is much less than the prediction for larger $G_h$.

Here, it is known that the elasticity dramatically increases with increasing the density near the close packing or jamming packing fraction. Thus *k* depends on the density after deformation, not constant. After the compression, $\rho ^{(h)}$ is slightly smaller than $\rho ^{(s)}$ ($\Delta \rho > 0$) and then this subtle density difference induces the large difference of the elasticity, that is, the large decrease of *k*. As a result, $\alpha$ decreases depended on $\Delta \rho$, or $E^{(h)}_{xx}$. This is consistent with the simulation results where the deviation of $\alpha$ from the theoretical prediction should be correlated with the slope of $u_x$ or $E^{(h)}_{xx}$ in the G$_h$ region.

## Conclusion

In summary, a simulation was performed in which a force was applied from above in a soft-particle model arranged in a triangular lattice pattern. We considered a system in which the softness of the particles was dispersed and the hardness of the internal region was non-uniform. The force distribution is localized in the harder region but it is largely deviated from the prediction from the continuum approximation. It is found that the nonlinear distribution strongly depends on the expansion of the harder region in *x* due to the repulsive interaction. Meanwhile, the force distribution becomes close to the theoretical prediction when the strength of the attractive interaction is similar to that of the repulsive interaction, that is, the potential is close to the symmetric.

In this time, we have clarified the most basic state, but we consider that the future work is to introduce quantitative control of roughness and friction and expand it to more realistic problems. When there is friction between particles, it is expected that the spatial distribution of displacement will be suppressed and the force propagation will change significantly. Furthermore, it is expected to be applied to more industrial fields by clarifying the mechanical properties of composite material systems such as dynamic behaviors when a force is applied locally as well as uniform pressure.
